# Editorial: Recent Advances in Pediatric Cancer Predisposition Syndromes

**DOI:** 10.3389/fped.2021.661894

**Published:** 2021-03-08

**Authors:** Angela Mastronuzzi, Luigi Boccuto, Riccardo Masetti

**Affiliations:** ^1^Department of Onco-Hematology, Cell and Gene Therapy, Bambino Gesù Children's Hospital (IRCCS), Rome, Italy; ^2^School of Nursing, College of Behavioral, Social and Health Sciences Healthcare Genetics Interdisciplinary Doctoral Program, Clemson University, Clemson, SC, United States; ^3^JC Self Research Institute, Greenwood Genetic Center, Greenwood, SC, United States; ^4^Pediatric Oncology and Hematology “Lalla Seràgnoli”, Pediatric Unit, IRCCS Azienda Ospedaliero-Universitaria di Bologna, Bologna, Italy

**Keywords:** cancer, predisposition, syndromes, pediatric, oncology, children

Cancer predisposition syndromes (CPSs) are an important cause of tumors in pediatric patients. Although a significant number of cancer predisposition genes have already been described, there are many pediatric patients with cancer in whom inherited cancer predisposition syndromes have yet to be detected.

The prevalence of childhood cancer attributable to genetic predisposition is difficult to be estimated but recent reports suggest that at least 10% of pediatric cancer patients harbor a germline mutation in a cancer-predisposition gene.

The advent of large-scale genome sequencing studies has profoundly helped our understanding of the biology of cancer predisposition, leading to better and earlier identification of individuals at high risk of cancer, selection of new molecular targets, and, in some cases, development of tailored approaches.

The Research Topic on “Recent Advances in Pediatric Cancer Predisposition Syndromes” included original contributions and reviews on different aspects of pediatric cancer predisposition syndrome.

Central nervous system tumors are the first cause of solid malignancies in children, and the leading cause of morbidity and mortality in young adults. Cancer predisposition syndromes are seen in children with brain tumors in much higher frequency than other childhood cancers. These syndromes predispose the individual and family members to multiple cancers in different sites. Recent genetic discoveries and careful observation and surveillance resulted in improved survival, reduced morbidity, and targeted therapies for these children. In some contributions of the present topic, the authors discuss clinical manifestations, genetic overview, and management of these complex syndromes in brain cancer.

Ceglie et al. described cancer predisposition syndrome in Pediatric High-Grade Gliomas (pHGG). In the review, the authors summarize the main pHGG-associated cancer predisposing disorders, suggesting indicationsfor suspecting these syndromes and referring for genetic counseling. Better understanding of pHGG-associated syndromes can not only help identify them more quickly and thus provide families with informative genetic counseling but can also lead to a broader knowledge of the tumor-specific genetic landscape and thus of the possible target therapies.

Medulloblastoma is the most frequent malignant brain tumor observed in infancy. Carta et al. presented a detailed overview of CPSs related to medulloblastoma, describing their clinical, epidemiological, genetic, diagnostic, and therapeutic features. Understanding the associations between cancer predisposition syndromes and the different molecular subgroups of medulloblastoma can guide the development of novel targeted therapies, helping to elucidate differences in prognosis and therapeutic vulnerability. This may also help to further improve surveillance measures, to ensure the best quality of care for these patients.

Rhabdoid tumor predisposition syndrome (RTPS) is a rare condition characterized by a high risk of developing rhabdoid tumors such as atypical teratoid rhabdoid tumors (AT/RT), mainly aggressive and multifocal cancers that arise mostly before 1 year of age. RTPS1 is characterized by pathogenic variants in the *SMARCB1* gene, while RTPS2 has variants in *SMARCA4*. Del Baldo et al. provided a wide clinical and genetic description of RTPS types 1 and 2. Moreover, the authors highlighted the importance of early diagnosis of RTPS with references to surveillance proposition, genetic tests, and counseling recommendations to family members. Further research is needed to increase our understanding of rhabdoid tumor biology and the role of *SMARCB1*/*SMARCA4* tumor development.

DICER 1 syndrome (DS) is a cancer-predisposing disorder caused by pathogenic variants in the *DICER1* gene that confer an increased risk to develop a neoplasm in childhood of about 5.3% before 10 years of age. Its pathognomonic feature is the pleuropulmonary blastoma (PPB), but cancer can arise in many other sites. Caroleo et al. provided a review on this interesting topic. According to the authors, screening for DS should always be performed in patients with PPB and should be considered in the presence of other specific benign and malignant lesions. Early identification of DS is essential for planning an adequate follow-up to manage the risk of cancer occurrence in carriers of pathogenic *DICER1* variants.

About rare conditions, Miele et al. examined clinical and genetic features of 13 children affected by pediatric adrenocortical tumors, very rare endocrine neoplasms. They described an excellent prognosis, with a 5-year overall survival of 100% and 5-year disease-free survival of 84.6%. In 75% of patients tested the *TP53* gene was mutated, supporting the indication for genetic testing and family counseling in this disease.

The contribution of Chiang et al. offers an overview of predictive testing for CPSs in pediatric relatives in Asian countries. They conducted a retrospective analysis including families with germline pathogenic/likely pathogenic variants identified in genes associated with pediatric cancer susceptibility and conclude that the rate of predictive testing in pediatric first-degree relatives (FDRs) is higher than that of adults in Asia, albeit below the global average. They hypothesize that factors that may influence the uptake of predictive testing in pediatric FDRs include a lack of information about genetics, preoccupations regarding health insurance, and genetic discrimination.

To note, any cancerous transformation can result from mutations inherited or acquired throughout life. In this scenario, DNA repair mechanisms are crucial to preserve genomic integrity. DNA repair syndromes with a biallelic disorder of essential DNA damage response pathways generally occur early in life by exposing to a high susceptibility to develop hematologic and solid tumors. Sharma et al. described classic biallelic DNA repair cancer syndromes arising from defective single- and double-strand DNA break repair, as well as dysfunctional DNA helicases, providing a historical overview and discussion about complex biology and heterogeneous clinical manifestations.

Concerning vascular tumors in pediatric patients, Hinen et al. described major vascular tumors in the pediatric population with reference to International Society for the Study of Vascular Anomalies (ISSVA) classification guidelines for vascular anomalies (2018). A detailed description of vascular tumors (benign, locally aggressive/borderline, and malignant) and vascular malformations highlighted the importance to recognize high-risk characteristics of each cancer, including anatomic risks, morphology, potential for the co-occurrence of congenital defects, coagulopathy, and malignant evolution.

Capasso et al. provided a very detailed description of genetic variants that predispose to pediatric solid tumors (neuroblastoma, Wilms tumor, retinoblastoma, ependymoma, medulloblastoma, astrocytoma, osteosarcoma, Ewing sarcoma, and rhabdomyosarcoma). They underlined the interactions between germline and somatic alterations as a determinant of cancer development and proposed future research directions focused on this and the importance to develop new molecular diagnostic tests.

As already known, overgrowth syndromes have been linked to an enhanced risk of cancer development and share key molecular pathways involved in cell growth and proliferation with several pediatric cancers. Griff et al. summarized the present data on cancer burden among these conditions and their associated cancer screening guidelines.

Cancer predisposition syndromes remain a challenging issue in pediatric cancer. The rapidly evolving scenario raises numerous biological, clinical, and ethical questions. Continuous efforts should be put into these issues by pediatric oncologists and hematologists in the near future. We believe that advancing knowledge in clinical and research fields would be important to improve the clinical outcome of patients.

## Author Contributions

All authors wrote and revised the editorial.

## Conflict of Interest

The authors declare that the research was conducted in the absence of any commercial or financial relationships that could be construed as a potential conflict of interest.

